# ERO1 alpha deficiency impairs angiogenesis by increasing N-glycosylation of a proangiogenic VEGFA

**DOI:** 10.1016/j.redox.2022.102455

**Published:** 2022-08-27

**Authors:** Ersilia Varone, Alexander Chernorudskiy, Alessandro Cherubini, Angela Cattaneo, Angela Bachi, Stefano Fumagalli, Gizem Erol, Marco Gobbi, Michael J. Lenardo, Nica Borgese, Ester Zito

**Affiliations:** a Istituto di Ricerche Farmacologiche Mario Negri IRCCS, Milan, Italy; b Proteomics/MS Facility, Cogentech SRL Benefit Corporation, Milan, Italy; c IFOM-FIRC Institute of Molecular Oncology, Milan, Italy; d Molecular Development of the Immune System Section, Laboratory of Immune System Biology, And Clinical Genomics Program, Division of Intramural Research, National Institute of Allergy and Infectious Diseases, National Institutes of Health, Bethesda, MD, USA; e CNR Neuroscience Institute, c/o University of Milano Bicocca, Vedano al Lambro (MB), Italy; f Department of Biomolecular Sciences, University of Urbino Carlo Bo, Italy

**Keywords:** ERO1 alpha, Oxidative folding, VEGFA, N-glycosylation, Angiogenesis, ERO1- alpha, Endoplasmic oxidoreductin 1-alpha, MAGT1, Magnesium transporter 1, OST, Oligosaccharyl transferase, VEGF, Vascular endothelial growth factor

## Abstract

N-glycosylation and disulfide bond formation are two essential steps in protein folding that occur in the endoplasmic reticulum (ER) and reciprocally influence each other. Here, to analyze crosstalk between N-glycosylation and oxidation, we investigated how the protein disulfide oxidase ERO1-alpha affects glycosylation of the angiogenic VEGF^121^, a key regulator of vascular homeostasis. ERO1 deficiency, while retarding disulfide bond formation in VEGF^121^, increased utilization of its single N-glycosylation sequon, which lies close to an intra-polypeptide disulfide bridge, and concomitantly slowed its secretion. Unbiased mass-spectrometric analysis revealed interactions between VEGF^121^ and N-glycosylation pathway proteins in ERO1-knockout (KO), but not wild-type cells. Notably, MAGT1, a thioredoxin-containing component of the post-translational oligosaccharyltransferase complex, was a major hit exclusive to ERO1-deficient cells. Thus, both a reduced rate of formation of disulfide bridges, and the increased trapping potential of MAGT1 may increase N-glycosylation of VEGF^121^. Extending our investigation to tissues, we observed altered lectin staining of ERO1 KO breast tumor xenografts, implicating ERO1 as a physiologic regulator of protein N-glycosylation. Our study, highlighting the effect of ERO1 loss on N-glycosylation of proteins, is particularly relevant not only to angiogenesis but also to other cancer patho-mechanisms in light of recent findings suggesting a close causal link between alterations in protein glycosylation and cancer development.

## 1 Introduction

Newly synthesized proteins released into the lumen of the endoplasmic reticulum (ER) are co- and post-translationally modified to reach a defined spatial conformation before being exported through the secretory pathway [1]. Both asparagine (N)-linked glycosylation and disulfide bond formation are two essential protein modifications occurring in the ER lumen [2,3]. These modifications can occur co-translationally, i.e., on the ribosome-associated nascent polypeptide, or post-translationally, i.e., after delivery of the completed polypeptide to the ER lumen [4,5]. Although N-linked glycosylation and disulfide bond formation are functionally independent because they rely on different structural features, respectively the triad Asn-X-Ser/Thr (where X is any amino acid other than proline) and cysteine, *de facto* the two processes can influence each other [6].

The Asn residue within the consensus acceptor site in proteins is glycosylated via the oligosaccharyl transferase (OST) complex. Metazoans express two OST catalytic subunits (STT3A and STT3B) which belong to two physically and functionally distinct complexes. Indeed, the STT3A complex promotes the co-translational N-glycosylation of substrate polypeptides, whereas the one containing STT3B mainly modifies sites that are skipped by STT3A, i.e. it works post-translationally [7].

Aside from a set of proteins associated with both OST complexes, additional accessory proteins are isoform-specific. This is the case, for example, of magnesium transporter 1 (MAGT1) and tumor suppressor candidate 3 (TUSC3), both associated with STT3B [8]. Interestingly, MAGT1 and TUSC3, which are both transmembrane proteins, share a lumenally-localized thioredoxin-like motif (CXXC) [9]: mutagenesis of this motif reduces glycosylation of STT3B substrates. Furthermore, N-glycosylation of both STT3A and STT3B substrates is favored by reducing conditions, suggesting a causal link between a more reduced ER redox poise and improved N-glycosylation [8,10].

Disulfide bond formation of proteins within the ER, which ultimately results in the oxidative poise of the ER, is catalysed by the couple Protein Disulfide Isomerase (PDI) and ER Oxidoreductin 1alpha (ERO1alpha, henceforth ERO1). While PDI directly introduces disulfide bonds into nascent proteins, ERO1 accepts electrons from the reduced PDI and moves them to molecular oxygen: this electron relay produces H_2_O_2_ as end product, with the concomitant regeneration of the oxidized forms of PDI and ERO1 [[11], [12], [13]]. In mammals, the role of ERO1 as protein disulfide oxidase is not essential, as its absence is compensated by other ER oxidases, such as Peroxiredoxin 4 (PRDX4) and Glutathione Peroxidase 7/8 (GPX7/8) [[14], [15], [16]]. Nonetheless, deficiency of ERO1 in mammals results in aberrant phenotypes, which are likely due to a delay in disulfide bond formation within newly synthesized proteins in the ER [[17], [18], [19]].

We have previously investigated the role of ERO1 on the release of angiogenic factors in triple-negative breast cancers [20]. We found that ERO1 deletion leads to reduced secretion of many angiogenic factors, and among these Vascular Endothelial Growth Factor A (VEGF^121^), resulting *in vivo* in decreased tumor angiogenesis and metastasis.

VEGF^121^ interacts with its receptor, mainly VEGF Receptor 2 (VEGFR2), as a disulfide-linked homodimer, where each protomer also contains three intrachain disulfides, important for the correct folding of the protein into a structure known as cysteine knot [21]. A unique N-glycosylation consensus lies within two of the intrachain disulfide bridges [22]. How the formation of the disulfide bridges affects the N-glycosylation process of VEGF^121^ has not been investigated so far.

Here, to investigate whether ERO1 affects protein N-glycosylation, we have exploited ERO1 knock-out cells expressing VEGF^121^. We show that ERO1 deletion delays disulfide bond formation, reinforces the interaction of VEGF^121^ with MAGT1 of the STT3B OST complex, and results in the full utilization of the N-glycosylation consensus site of the growth factor. Surprisingly, this N-hyper-glycosylation causes a reduction in VEGF^121^ secretion. Our findings reveal an unanticipated role of ERO1 in regulating N-glycosylation and add a novel layer to the complexity of protein post-translational modifications and folding within the ER lumen.

## 2 Materials and methods

### 2.1 Cell culture and transfection

HeLa cells were transfected with ERO1-Lα CRISPR-Cas9 KO plasmids (Santa Cruz Biotechnology) with three target-specific guide RNAs (gRNA) of 20 nt. Clones were analyzed by SDS-PAGE and Sanger sequencing [23]. MAGT1-HA-pCI-neo vector was described in Ref. [24].

### 2.2 Hypoxic chamber

Cells were transferred to hypoxic chamber (Ruskinn Invivo2 400, UK) at 37°C and maintained in deoxygenated culture medium at the following gas concentrations: O_2_ 0.1%, CO_2_ 5% for 48 h. Control cells were maintained in standard culture medium in a normoxic incubator.

### 2.3 Cycloheximide (CHX) washout

Cells were transfected with the respective constructs using FuGENE HD transfection reagent (E2311, Roche) at a 1:3 DNA to reagent ratio; 24 h after transfection the cells were split into 6 cm Petri dishes in complete growth medium with 10% FBS. The next day subconfluent cells were exposed to CHX at 50 μg/mL in medium with 1% FBS for 6 h. After removal of the CHX, cells were left in the fresh medium (1% FBS) and collected at different times. Pelleted cells were washed with cold PBS, NEM 20 mM. The medium was also collected, supplemented with NEM (20 mM) and a protease inhibitor cocktail (11697498001 or 11873580001, Roche).

### 2.4 Small interfering RNA

Cells were plated in 6 cm Petri dishes at 400.000 cells/dish density and left to grow in complete DMEM medium with 10% FBS for 24 h before the first round of transfection with siRNA constructs. For the transfection, we used FuGENE HD transfection reagent (E2311, Roche) at a 1:3 DNA to reagent ratio in Opti-MEM I reduced serum medium (31985062, Gibco). Expression constructs for siRNA targeting human STT3B (EHU051881) or MAGT1 (EHU110431) were purchased from Sigma-Aldrich. Two rounds of transfection with 2 ug siRNA per 6 cm dish were run 24 h apart. The medium with 1% FBS was added to the cells 24 h after the second transfection and left for 16 h. Cells and medium were collected as described for CHX washout. One half of the cell pellet was dissolved in QIAzol lysis reagent (79306, Qiagen) and frozen at -80°C for subsequent RNA extraction. Another half was frozen as dry pellet for subsequent lysis and Western blot analysis.

### 2.5 Immunoprecipitation

Protein samples were lysed in cold RIPA buffer supplemented with 10 mM calcium and 20 mM NEM and a protease inhibitor cocktail, centrifuged at 16.100 g for 10 min to remove insoluble material and quantified by BCA assay (23227, Pierce). Samples containing 0.5–1 mg of total protein were pre-cleared using SureBeads protein G magnetic beads (1614023, Bio-Rad Laboratories) for 1 h and incubated with 20 uL of anti-FLAG M1 agarose affinity gel (A4596, Millipore) for 16 h at 4°C. Beads were than washed 3 times with 1 mL of cold lysis buffer, and immunoprecipitated proteins were eluted from the beads by heating to 70°C for 5 min in 2x non-reducing Laemmli buffer.

### 2.6 Western blotting and quantification

Protein concentration was determined with a standard BCA assay (Pierce). Samples with the same protein concentration were mixed with non-reducing Laemmli buffer (62.5 mM Tris-HCl pH 6.8, 2% SDS, 10% glycerol and 0.01% bromophenol blue) and heated for 5 min at 95°C. For reducing SDS-PAGE, samples were supplemented with 100 mM DTT. Protein samples separated by either reducing or non-reducing SDS-PAGE were then transferred to Protran nitrocellulose membrane (GE10600002, Amersham Protran, pore size 0.45 μm) and probed with the following antibodies: monoclonal mouse anti-Actin (MAB1501, Sigma Aldrich), monoclonal anti-FLAG M2 antibody produced in mouse (F3165, Sigma-Aldrich), monoclonal anti-HA antibody (6E2, Cell Signaling), polyclonal rabbit anti-ERO1 alpha [17] and polyclonal MAGT1 antibody (PA5-106315, Invitrogen), MAGT1 antibody (produced in house by the NIH/Merck collaborative project). The fluorescent secondary antibodies IRDye 680RD goat anti-mouse IgG (926–68070, Li-Cor) and IRDye 800CW goat anti-rabbit IgG (926–32211, Li-Cor) were used for protein detection. The fluorescent signal was acquired on a ChemiDoc MP Imaging System and quantified by Image Lab analysis software (Bio-Rad Laboratories).

### 2.7 Protein pathway and network analysis

To rank enriched terms EnrichR was used considering the Gene Names of the specific proteins identified in the samples of ERO1 KO; the enriched pathway - GO Biological Process is shown in Fig. 6A. Proteomic data were deposited in PRIDE (PXD033941).

### 2.8 Breast tumors

Eight-to ten-week-old female SCID mice were obtained from Charles River Laboratories (Calco, Italy) and maintained under specific-pathogen-free conditions. SCID mice were housed in isolated vented cages, and handled using aseptic procedures. Procedures involving animals were conducted in conformity with the following laws, regulations and policies governing the care and use of laboratory animals: Italian Governing Law (D.lgs 26/2014, authorization number19/2008-A issued 6 March 2008 by Ministry of Health; authorization 395/2018 PR to E.Zito); Mario Negri Institutional Regulations and Policies providing internal authorization for people conducting animal experiments (Quality Management System Certificate—UNI EN ISO9001: 2008—registration number 6121); EU directives and guidelines (EEC Council Directive 2010/63/UE), and in line with Guidelines for the welfare and use of animals in cancer research [25].

Mice were inoculated with WT- and ERO1 KO-MDAMB231^m^ in the mammary fat pad, and sacrificed between 30 and 45 days respectively when the tumor reached a size of approximately 100–150 mm^3^ [20]. Frozen tumors were cut in 10 μm sections, which were decorated with fluoconjugated Alexa 488 WGA (1 μg/mL, W11261, Molecular Probes), and Alexa 647 IB4 (2.5 μg/mL, Thermofisher) and Hoechst to label nuclei. Interference of autofluorescence was excluded by the absence of signal in sections not exposed to lectins (Fig. Sup. 6). Confocal microscopy was done using a sequential scanning mode to avoid bleed-through effects with a FV500 Olympus confocal microscope. Three fields of view sized 240 x 180 × 7 μm were acquired with a 0.9 μm step size for each section. Fields of view were randomly positioned where a coronal cut vessel was identified based on the Hoechst signal. Microphotographs were taken with an oil immersion 60x objective with a pixel size of 0.3 μm, and processed by ImageJ. Briefly background was corrected using ImageJ’s subtract background with a 20 pixel rolling ball. Intensity of WGA- and IB4-stained sections was calculated on the mean gray values along the z stack and expressed as integrated density. One of the five samples of the WT was identified as an outlier by a Grubb’s test with alpha at 0.05 and thus excluded from the analysis. Because the signal in WGA-stained sections was clustered, we analyzed the percent volume occupied by these clusters: we generated the extended focus image on maximum pixel intensity, and ran trainable Weka segmentation to identify WGA clusters. A probability image was then created and only the signal with >90% of probability of corresponding to clustered WGA was quantified. The area fraction (% area) occupied by the positive signal was calculated and used for statistical analysis.

### 2.9 Statistics

Data are the mean ± SEM, ± SD in Fig. 7, and were analyzed with Prism 7 (Graphpad). N is indicated in the figure legends. Statistical significance was assessed with the unpaired *t*-test for two-group analysis and one-way ANOVA or two-way ANOVA for multiple comparisons. For Fig. 7 we considered the mean area fraction of the three fields of view per sample (N = 5) and all data passed the Kolmogorov-Smirnov test for normality. One, two, three, and four symbols indicate p < 0.05, p < 0.01, p < 0.001 and p < 0.0001, respectively.

## 3 Results

### 3.1 Diminished oxidative poise and impaired oxidative bond formation in ERO1 knock-out cells

**Fig. 1:**
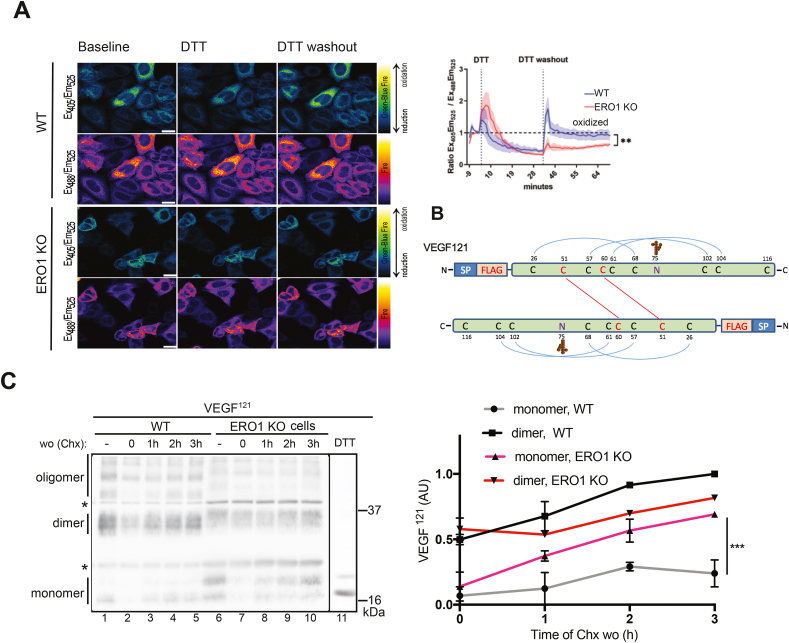
Impaired oxidative poise and delay in disulfide bond formation of VEGF^121^ in ERO1 KO cells Fluorescent photomicrographs of WT and ERO1 KO cells expressing an ER-localized roGFP2 (obtained at two different excitation wavelengths, namely 405 or 488 nm). On the right, time-dependent changes in the fluorescence excitation ratio of roGFP2, reflecting the alterations in the redox state of roGFP2 localized in the ER of WT and ERO1 KO HeLa cells after a DTT pulse of 30 min followed by a washout period of the reducing agent (N = 4 randomly selected fields of view each having 9–16 cells; solid and dotted lines are mean values and SEM respectively; two-way ANOVA) (scale bar 20 μm). B) Scheme of FLAG-VEGF^121^ protein. The signal peptide (SP), the FLAG tag, the cysteines (C) involved in intrachain and interchain disulfide bonds and the glycosylated asparagine (N75) are indicated. C) Non-reducing immunoblot of FLAG-VEGF^121^ immunoprecipitates extracted from WT and ERO1 KO HeLa cells. The cells were exposed to a 6 h pulse of the protein synthesis inhibitor CHX followed by a washout for the indicated times. The lanes marked with a minus symbol (lanes 1 and 6) contain samples of cells before application of the CHX pulse. The last lane (11) contains a sample from WT cells reduced with DTT, which indicates the FLAG-VEGF^121^ monomer. The asterisks indicate IgG light and heavy chains. The migration of monomer, dimer and oligomer of VEGF^121^ are noted, and the relative distribution of monomer and dimer during CHX washout depicted graphically in the panel on the right (N = 3, two-way ANOVA).

To track ER redox poise in WT and ERO1 KO HeLa cells, we exploited the ratiometric redox sensor ER-localized roGFP2 [26,27]. The redox changes of this sensor in live cells after exposure to the reductant dithiothreitol (DTT) were measured by comparing sensor emission intensity at 525 nm when excited at 405 (Ex_405_Em_525_) and 488 (Ex_488_Em_525_) nm. The baseline redox signal of the ratio between Ex_405_Em_525_ and Ex_488_Em_525_ was set at 1; thus signals <1 or >1 indicated the reduction or oxidation of the sensor compared to baseline respectively. As shown in Fig. 1A, ER roGFP2 was rapidly reduced after a challenge with DTT in both WT and ERO1 KO cells; however, during washout from the reductant, ERO1 KO cells exhibited a slower recovery: whereas the oxidative poise of WT cells recovered rapidly, and in the 40 min washout period never reached the baseline value of oxidative poise of WT, suggesting that a lack of ERO1 impairs ER oxidative poise [28].

To assess the impact of ERO1 loss on the rate of disulfide bond formation in the ER, we exploited a FLAG-VEGF^121^ construct, which is assembled into a disulfide-linked homodimer [22] (Fig. 1B), and compared the rate at which disulfide-linked homodimers form intracellularly after a pulse of the inhibitor of protein synthesis cycloheximide (CHX) in WT and ERO1 KO cells. After washout from CHX, FLAG-VEGF^121^ accumulated to a similar extent in WT and ERO1 KO cells; however, whereas in WT cells the dimer was the main species at all time points, in ERO1 KO cells generation of the dimer was slower and the monomer accumulated during the washout, indicating a slower conversion of the monomers into dimers (Fig. 1C, Sup. Fig. 1). Thus, the alteration in oxidative poise caused by loss of ERO1 translates in an impairment of the rate of VEGF^121^ disulfide bond formation.

### 3.2 VEGF^121^ secreted by ERO1 KO cells does not display a defect in intrachain disulfide bonds but hyperglycosylation on N75

**Fig. 2:**
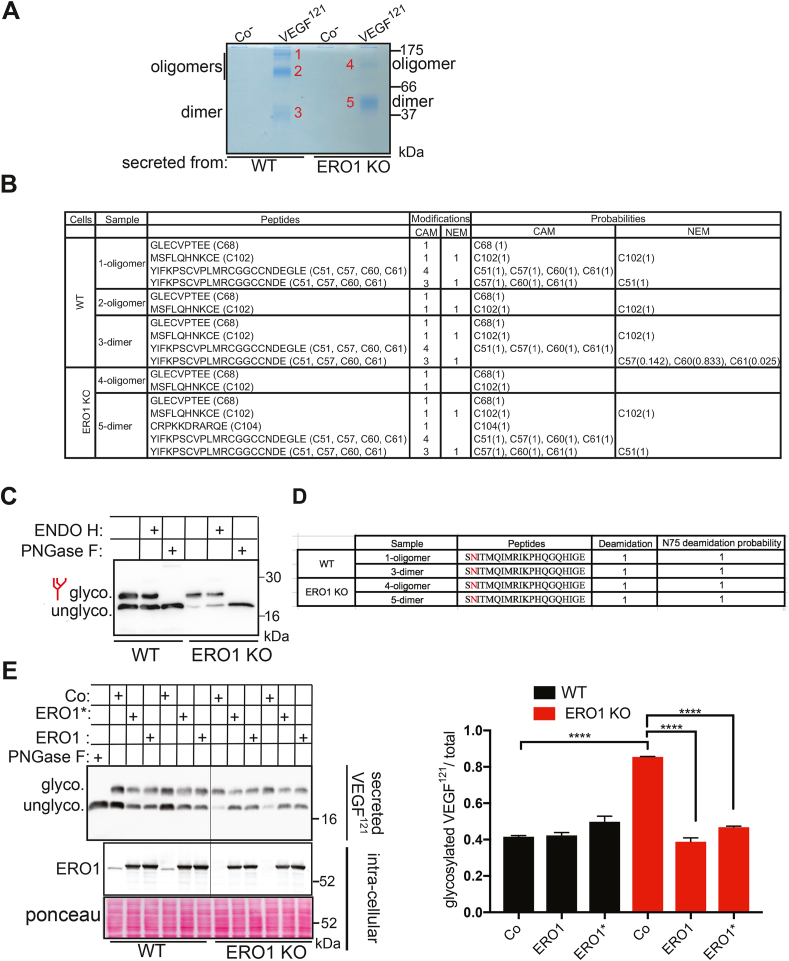
Native intrachain disulfide bonds and N-hyperglycosylation in VEGF^121^ secreted from ERO1 KO cells A) Coomassie stained non-reducing SDS-PAGE of FLAGM1-immunopurified VEGF^121^ secreted from WT and ERO1 KO cells (Co^−^ indicates FLAGM1 immunoprecipitates from lysate of cells transfected with an empty vector). The bands corresponding to the dimeric and oligomeric forms (assigned on the basis of their apparent molecular weights and of the migration of the monomer in reducing gels -see Sup. Fig. 2A) were excised and the redox state of cysteines was analyzed by nLC-ESI-MS/MS sequence analysis. The numbers in red to the right of each band identify the samples reported in the table of panel B. B) Table reporting the redox state of cysteines (the position of each cys residue is reported within parentheses) in VEGF^121^ dimer and oligomer as from the numbers given in panel A. NEM and Carbamidomethyl (CAM) modifications report on reduced and oxidized cys residues, respectively; the probability of different alkylation types (derived from MaxQuant analysis) is reported in the last column. C) FLAG Immunoblot of secreted VEGF^121^ after EndoH and PNGaseF treatment, glyco. (glycosylated), unglyco. (unglycosylated). D) Table reporting deamidation of FLAG-VEGF^121^ as in A after PNGaseF treatment. The N75 residue is highlighted in red. E) FLAG Immunoblot of VEGF^121^ secreted from WT and ERO1 KO cells following transfection of a plasmid vector encoding ERO1 or its hyperactive mutant C131A, indicated as ERO1*. Co: control cells transfected with empty vector. The first lane shows a PNGase-digested sample as size marker for the unglycosylated polypeptide. The black vertical line is drawn over the blot to facilitate distinction of the group of samples from WT and ERO1 KO cells. The bottom panel shows an ERO1 blot of lysates of the same cells. Ponceau staining shows the equal protein loading. The bar graph on the right shows the ratio between glycosylated VEGF and the total (unglycosylated + glycosylated) under the indicated conditions (N = 3, one-way ANOVA). (For interpretation of the references to colour in this figure legend, the reader is referred to the Web version of this article.)

To analyze FLAG-VEGF^121^ secreted from WT and ERO1 KO HeLa cells, conditioned media from the two cell lines were collected, alkylated with N-ethylmaleimide (NEM), and then immunoprecipitated with FLAG-M1 antibody. The immunoprecipitates were analyzed by non-reducing SDS-PAGE. Coomassie staining of the gel revealed three main bands of FLAG-VEGF^121^ from WT cells, corresponding to dimeric and oligomeric species (based on their molecular weights) of VEGF^121^ while FLAG-VEGF^121^ from ERO1 KO cells was detectable only in two bands, i.e. a faint oligomer and a dimer migrating more slowly than the one secreted from WT cells (Fig. 2A and Sup. Fig. 2A). These bands were excised from the gels, DTT-reduced and alkylated with iodoacetamide (IAA), a treatment which led to labeling of the cysteines involved in disulfide bonds resulting in the addiction of a carbamidomethyl group (CAM), and finally subjected to nLC-ESI-MS/MS (Fig. 2B). The mass spectra of the fragmented peptides showed no major difference in NEM and IAA peptide alkylation between the FLAG-VEGF^121^ secreted from WT or from ERO1 KO. For example, C68, which is involved in an intrachain disulfide bond in VEGF^121^ is present only in its oxidized form in VEGF^121^ from both WT and ERO1 KO cells. C102 is also involved in an intrachain disulfide bond in VEGF^121^ and is detected in a mixed reduced and oxidized state in VEGF^121^ from both WT and ERO1 KO cells, suggesting no defective intramolecular disulfide bonds of FLAG-VEGF^121^ from ERO1 KO cells (Fig. 2B) [22].

Analysis by reducing SDS-PAGE-immunoblot, showed that secreted FLAG-VEGF^121^ migrates in two major bands, whose relative ratios differed between WT and ERO1 KO cells, i.e. the slower migrating band was more abundant in VEGF^121^ from ERO1 KO cells. Examination of the VEGF^121^ amino acid sequence using the bioinformatics program NetNGlyc (https://services.healthtech.dtu.dk/service.php?NetNGlyc-1.0) predicts the presence of a putative N-glycosylation site at Asn in position (N75) within the consensus sequence NIT. Indeed, N-glycosylation of VEGFA has been reported, although its functional significance remains unclear [29]. We therefore hypothesized that the slower migrating band was a product of N-glycosylation and decided to test this with the use of endoglycosidases.

As shown in Fig. 2C, FLAG-VEGF^121^ from both WT and ERO1 KO cells was insensitive to the endoglycosidase H (ENDO H), but sensitive to the peptide-N-glycosydase F (PNGase F), resulting in collapse of the protein in the faster migrating species. These results demonstrate that indeed the slower migrating polypeptide contains N-linked oligosaccharide that has undergone maturation in the Golgi apparatus, thereby acquiring EndoH resistance.

Upon treatment with PNGase F (which is an amidase), glycosylated Asn residues are deamidated. The mass spectra of the fragmented peptides of the dimer and oligomers of FLAG-VEGF^121^ from WT and ERO1 KO cells and then digested with PNGaseF confirmed that the Asparagin N75 was glycosylated (identified as aspartic acid upon PNGase F digestion [30]), except for the oligomeric species 2 derived from WT cells (Fig. 2D and Sup. Fig. 2B). The role of ERO1 in limiting glycosylation was confirmed by rescue experiments. Indeed, transfection of an expression plasmid driving ERO1 or its hyperactive mutant ERO1^C131A^ in ERO1 KO cells, rescued the N-hyperglycosylation of FLAG-VEGF^121^ secreted from ERO1 KO cells, by shifting the glycosylated form (calculated as the glycosylated form on the total glycosylated and unglycosylated) from 80% to 40% of the total (Fig. 2E).

### 3.3 Hyperglycosylation of N75 in VEGF^121^ of ERO1 KO cells causes defective kinetics of secretion

**Fig. 3:**
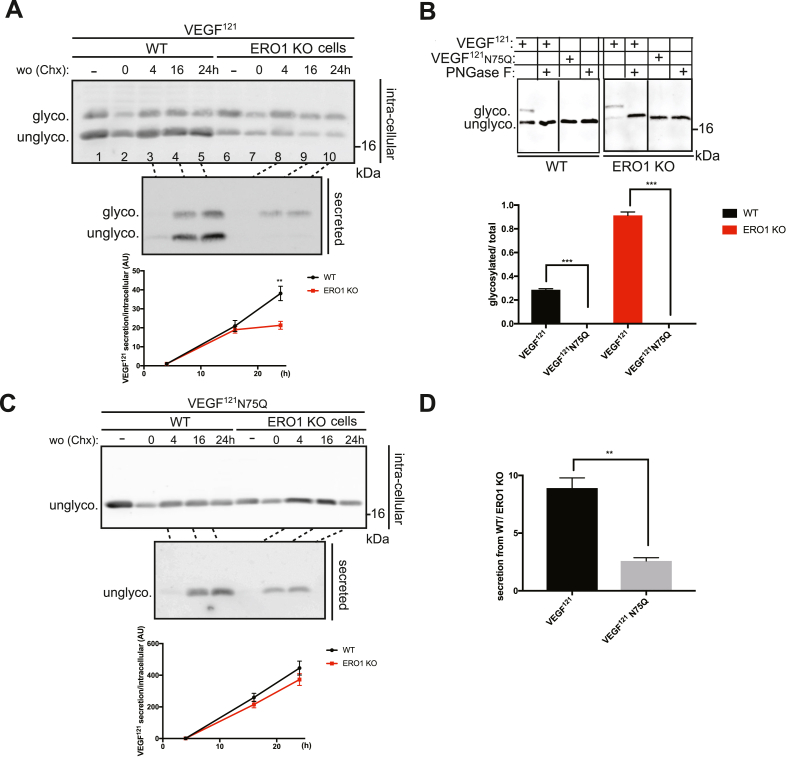
N75 hyper-glycosylation in VEGF^121^ secreted from ERO1 KO impairs its kinetics of secretion A) Reducing immunoblot of intracellular and secreted FLAG-VEGF^121^ from WT and ERO1 KO HeLa cells. The cells were exposed to a pulse of the protein synthesis inhibitor CHX followed by a washout for the indicated times. The lane marked with a minus symbol (lanes 1 and 6) contain samples of cells before application of the CHX pulse. The amount of VEGF121 secretion relative to intracellular VEGF121 is depicted graphically in the panel below the blot (N = 3, two-way ANOVA). B) Reducing FLAG Immunoblot of FLAG-VEGF^121^ and N75Q secreted from WT and ERO1 KO cells. The slowly migrating band of FLAG-VEGF^121^ disappears completely in the N75Q mutant. The vertical black line in the two panels indicates the removal of irrelevant lanes of the blot in the image (see Fig. Sup. 3 for the uncropped blot). The bar graph below the blot shows the ratio between glycosylated VEGF and the total (N = 3, *t*-test). C) Kinetics of secretion of the N75Q FLAG-VEGF^121^ mutant (N = 3, two-way ANOVA). Experimental conditions were as described for panel A. D) Bar graph indicating FLAG-VEGF^121^ secretion from WT cells at the three washout points of 4, 16 and 24h over the correspondent from ERO1 KO cells (N = 3, *t*-test).

We next compared the kinetics of VEGF^121^ secretion between WT and ERO1 KO cells and investigated how N-hyper-glycosylation might affect this process. A pulse of CHX in FLAG-VEGF^121^-transfected WT and ERO1 KO cells followed by different chase times served to analyze the kinetics of secretion of FLAG-VEGF^121^ (secreted/intracellular) and revealed impaired kinetics of secretion of VEGF^121^ from ERO1 KO cells, which reaches a plateau at the last two time points (Fig. 3A and Fig. Sup. 3A). To investigate whether the impairment was due to hyper-glycosylation, we generated a mutant, in which N75 was substituted by a Gln residue (N75Q). As expected, this mutant of VEGF^121^ was not glycosylated when expressed either in WT and in ERO1 KO cells (Fig. 3B and Sup. Fig. 3B). Remarkably, the kinetics of secretion of this mutant were comparable in WT and ERO1 KO cells and the secretion was less affected in ERO1 KO cells (Fig. 3C, Fig. Sup. 3C and Fig. 3D), suggesting that the hyper-glycosylation of N75 underlies the impaired secretion of FLAG-VEGF^121^ in ERO1 KO cells.

### 3.4 VEGF^121^ from ERO1 KO cells is still receptor competent but the receptor competency is lost under hypoxia

**Fig. 4:**
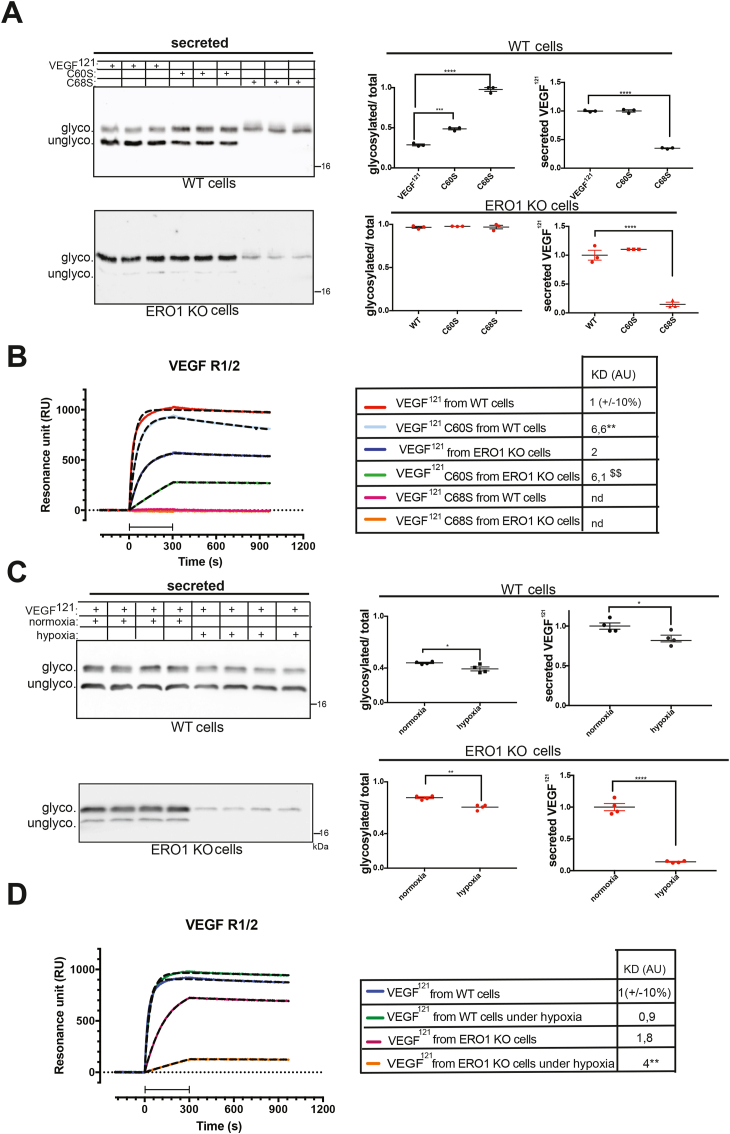
VEGF^121^ from ERO1 KO cells is receptor competent but its receptor competency is impaired under hypoxic conditions A) FLAG Immunoblot of native and cysteine mutant VEGF^121^ forms secreted from WT and ERO1 KO cells. The percentage of glycosylated over total VEGF^121^ and the percentage of secretion of the two cysteine-mutant VEGF^121^ forms compared to VEGF^121^ are shown in the two graphs on the right side of the panel (N = 3, One-way Anova). B) Sensorgrams (in Resonance units, RU) of VEGF^121^ signal obtained by flowing conditioned media for 300 s (bar) on immobilized Aflibercept (containing VEGFR1 and R2). On the right, tables with KD in arbitrary units (AU), with the value for native VEGF^121^ set at one. ** extra-sum-of-squares F test vs VEGF^121^ from WT cells, $$ extra-sum-of-squares F test vs VEGF^121^ from ERO1 KO cells. C) FLAG Immunoblot of VEGF^121^ secreted from WT and ERO1 KO cells under normoxic and hypoxic conditions. The percentage of glycosylated over total VEGF^121^ and the percentage of secreted VEGF^121^ under hypoxic conditions and compared to that in normoxic conditions are shown in the graphs on the right side of the panel (N = 4, *t*-test). D) Sensograms (in Resonance units, RU) of VEGF^121^ signal and on the right, table with the KDs in AU, as in panel B. ** extra-sum-of-squares F test vs VEGF^121^ from ERO1 KO cells.

Mutagenesis into serine of C60 (C60S) or C68 (C68S) of FLAG-VEGF^121^, which are involved respectively in an inter-chain and an intra-chain disulfide bond (Fig. 1B), increased the N-glycosylation of VEGF^121^ secreted from WT cells, indicating that impairment of disulfide bond formation in VEGF improves its N-glycosylation (Fig. 4A, upper panel). The secretion of C60 from WT cells was not affected when compared to that of the un-mutated VEGF^121^ while the secretion of C68 was impaired of 60% (Fig. 4A, upper panel). The effect of hyper-glycosylation was not observed when the two mutants C60 and C68 were secreted from ERO1 KO cells, indicating a dominant effect of ERO1 loss on VEGF^121^ glycosylation (Fig. 4A, lower panel). The secretion of C60 from ERO1 KO cells was not affected when compared to that of the un-mutated VEGF^121^ from the same cells while the secretion of C68 was impaired of 85%, even more dramatically of C68 from WT cells (Fig. 4A, lower panel).

We exploited surface plasmon resonance (SPR) to determine the relative dissociation constant (KD), a measure of affinity, of different VEGF^121^ forms for Aflibercept, which is a fusion protein between an Fc region of human IgG1 and the two receptors of VEGF, VEGFR1 and R2 [31]. The results indicated that VEGF^121^ secreted from ERO1 KO cells had roughly two times less affinity for its receptors than the form secreted from WT cells (albeit without reaching a statistically significant difference), but that the affinity of mutant VEGF^121^ C60S was six times less while the mutant VEGF^121^ C68S did not bind the receptors at all when compared to VEGF^121^ secreted from WT cells. These results suggest that VEGF^121^ from ERO1 KO cells is still receptor-competent and rule out its massive oxidative unfolding (Fig. 4B).

Hypoxia is a condition of low oxygen concentration, which preferentially impairs post-translational disulfide bond formation [32]. Given the interplay between VEGF^121^ disulfide bond formation and N-glycosylation, we investigated the effect of hypoxia on VEGF^121^ secretion and glycosylation. VEGF^121^ secreted from WT cells under hypoxia showed negligible differences in terms of glycosylation or amount of secretion, when compared to the counterpart secreted under normoxia (Fig. 4C, upper panel). In addition, its KD was quantitatively similar to its counterpart secreted under normoxic conditions (Fig. 4D). In contrast, the amount of VEGF^121^ secreted from ERO1 KO cells was severely affected (of 85%) under hypoxia (Fig. 4C, lower panel), suggesting that under conditions of ERO1 loss VEGF^121^ maturation and secretion is particularly reliant on oxygen. Furthermore, a two-times lower KD suggests a further impairment of the receptor competency of VEGF^121^ from ERO1 KO cells under hypoxia (Fig. 4D).

### 3.5 Reinforced association between VEGF^121^ and STT3B leads to hyper-glycosylation in ERO1 KO cells

**Fig. 5:**
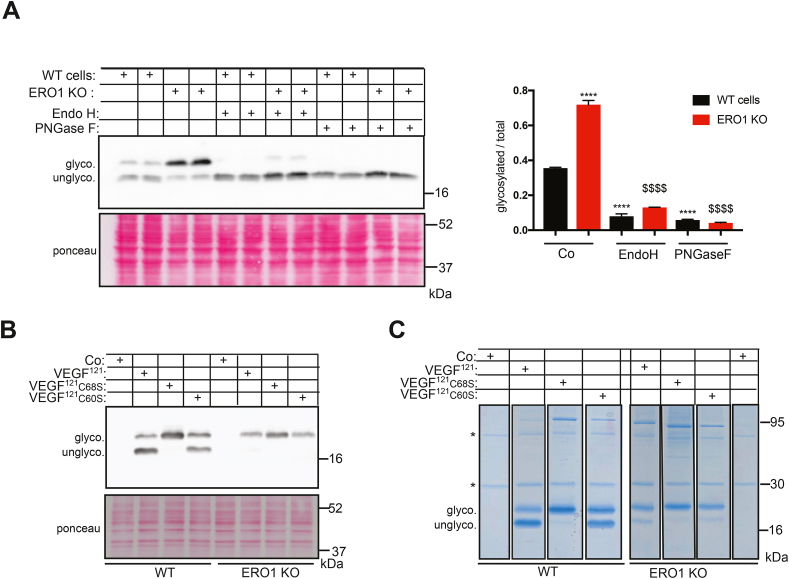
Intracellular VEGF^121^ in ERO1 KO and cysteine mutants are hyperglycosylated A) FLAG Immunoblot of VEGF^121^ in WT and ERO1 KO cells after EndoH and PNGaseF treatment. Ponceau staining shows the equal protein loading. On the right, bar graph indicating the ratio between glycosylated VEGF and the total. Co indicates cells not treated with glycosidases. Asterisks and dollar signs refer to comparison with VEGF^121^ in WT cells and ERO1 KO cells, respectively (N = 3, one-way ANOVA). B) FLAG Immunoblot of intracellular VEGF^121^, VEGF^121^C60S and C68S. Ponceau staining shows the equal protein loading. Co indicates cells transfected with an empty plasmid. C) Coomassie-stained reducing SDS-PAGE of FLAGM1-immunopurified VEGF121, VEGF^121^C60S and C68S in WT and ERO1 KO cells. The baits and the whole lanes were cut and analyzed for N-glycosylation and interactors.

As already observed, intracellular VEGF^121^, as well as the secreted form was hyper-glycosylated in ERO1 KO cells. However, differently from secreted VEGF^121^, the N-glycosylated intracellular form was sensitive to both EndoH and PNGaseF (Fig. 5A). No difference was observed between the sensitivity of VEGF^121^ to the two endoglycosidases in both WT and ERO1 KO cells, suggesting that, at steady state, most of the intracellular VEGF^121^ has not transited through the Golgi in both cell lines. Intracellular FLAG-VEGF^121^C60S and C68S behaved very similarly to the secreted counterparts being hyperglycosylated in WT cells and without relevant differences with respect to the native form in ERO1 KO (Fig. 5B).

To gain molecular insight into the causes and/or consequences of VEGF^121^ hyperglycosylation, we analyzed the interactors of VEGF^121^ in WT and ERO1 KO cells. Protein lysates from WT and ERO1 KO cells transfected with VEGF^121^, VEGF^121^C60S and VEGF^121^C68S were immunoprecipitated with FLAG M1 antibody, run on a reducing SDS-PAGE and stained with Coomassie (Fig. 5C and Sup. Fig. 4A). The staining confirmed VEGF^121^ hyper-glycosylation in ERO1 KO cells. The bands corresponding to the glycosylated and unglycosylated VEGF^121^ were excised and treated with PNGaseF and in-gel tryptic digestion; the eluted peptides were subjected to nLC-ESIMS/MS sequence analysis, confirming N75 as the only glycosylated asparagine also for the intracellular VEGF^121^ (Sup. Fig. 4B).

**Fig. 6:**
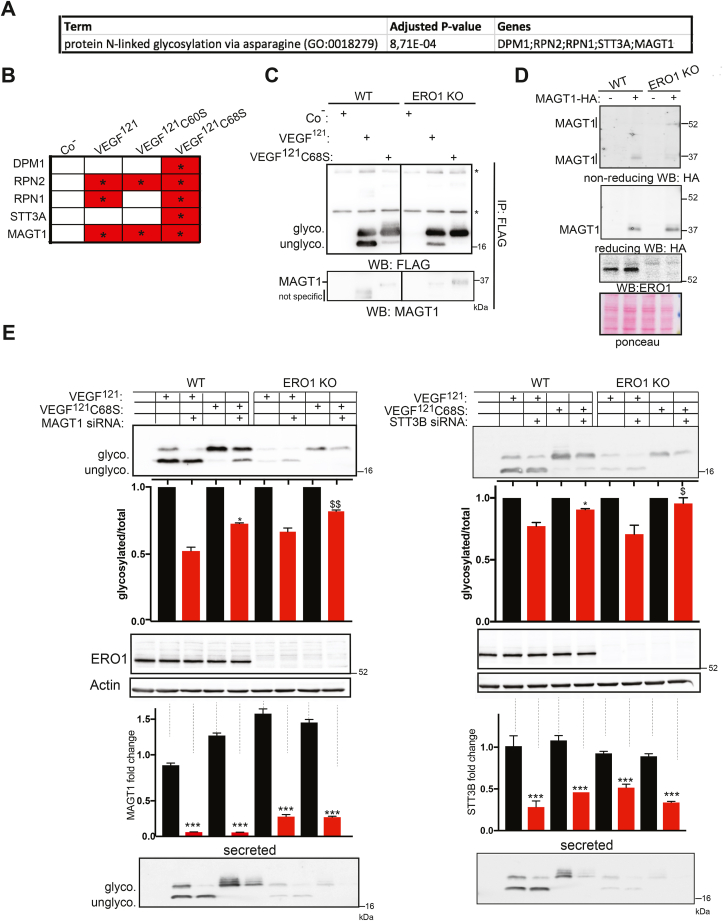
Enhanced interaction of VEGF^121^ and its cysteine mutants with MAGT1 in ERO1-KO cells A) GO terms of VEGF121, VEGF^121^C60S and C68S interactors in ERO1 KO cells that were not detected with the counterparts in WT cells. B) Tables of the indicated interactors of VEGF^121^, VEGF^121^C60S and VEGF^121^C68S in ERO1 KO cells belonging to the pathway of protein N-linked glycosylation via asparagine. None of the indicated proteins were detected in the VEGF^121^ pulldowns from WT cells. C) FLAG and MAGT1 immunoblot of FLAGM1-immunopurified VEGF^121^ and C68S in WT and ERO1 KO cells. The asterisks indicate the Ig light and heavy chain. The vertical black line in the two panels indicates the removal of irrelevant lanes of the blot in the image (see Fig. Sup. 6 for the uncropped blot). D) Non-reducing and reducing HA Immunoblot in MAGT1-HA transfected WT and ERO1 KO cells to detect a difference in MAGT1-HA redox state. - indicates cells transfected with an empty plasmid. MAGT1 appears as a doublet at 37 kDa in WT and ERO1 KO cells in reducing immunoblot while the predominant band is at 52 kDa in ERO1 KO cells in non-reducing immunoblot indicating a redox-dependent species of MAGT1. Bottom, ERO1 Immunoblot and ponceau. E) FLAG Immunoblot of intracellular VEGF^121^ and VEGF^121^C68S after MAGT1 and STT3B siRNA with the bar graphs indicating the glycosylated VEGF^121^ relative to the total. Asterisks and dollar signs refer to comparison with VEGF^121^ in WT cells and ERO1 KO cells, respectively (N = 3, *t*-test). Bottom, ERO1, Actin immunoblot and bar graphs indicating fold changes of MAGT1, STT3B cDNA, determined by q-PCR, for the intracellular counterparts (N = 3, *t*-test) and FLAG immunoblot of secreted VEGF^121^.

A series of other bands throughout the lanes were visible in the Coomassie-stained gels (Fig. 5C). Each lane was excised and underwent in-gel tryptic digestion; the eluted peptides were subjected to nLC-ESI MS/MS sequence analysis, leading to the identification of putative VEGF^121^ interactors. Pathway analysis of VEGF^121^, VEGF^121^C60S and VEGF^121^C68S interactors in ERO1 KO cells indicated enrichment of the pathway of the N-linked glycosylation via asparagine (GO terms) (Fig. 6A). Strikingly, the proteins belonging to the N-glycosylation pathway and associated with all three VEGF^121^ in ERO1 KO cells were not found at all associated with their counterparts in WT cells. Interestingly, MAGT1, a thioredoxin domain-containing subunit of the STT3B complex [8], was found to interact with all three VEGF^121^ in ERO1 KO cells (Fig. 6B). At this point we decided to focus on VEGF^121^C68S because holds mutation in C68 which is the closest to the glycosylated Asn and as such, whose glycosylation should be less influenced by MAGT1 [8]. Immunoprecipitates of VEGF^121^ and VEGF^121^C68S in WT and ERO1 KO cells confirmed a MAGT1 immunoreactive band in both VEGF^121^ and VEGF^121^C68S of ERO1 KO cells (Fig. 6C and Sup. Fig. 5). NEM alkylation of WT and ERO1 KO cells transfected with MAGT1-HA allowed resolution of the different redox forms of MAGT1 on a non-reducing Immunoblot. MAGT1 from ERO1 KO cells appeared in a slow migrating band around 52 kDa, which was not visible in that from WT cells and indicates a redox-mediated interaction of MAGT1 in ERO1 KO cells (Fig. 6D).

**Fig. 7:**
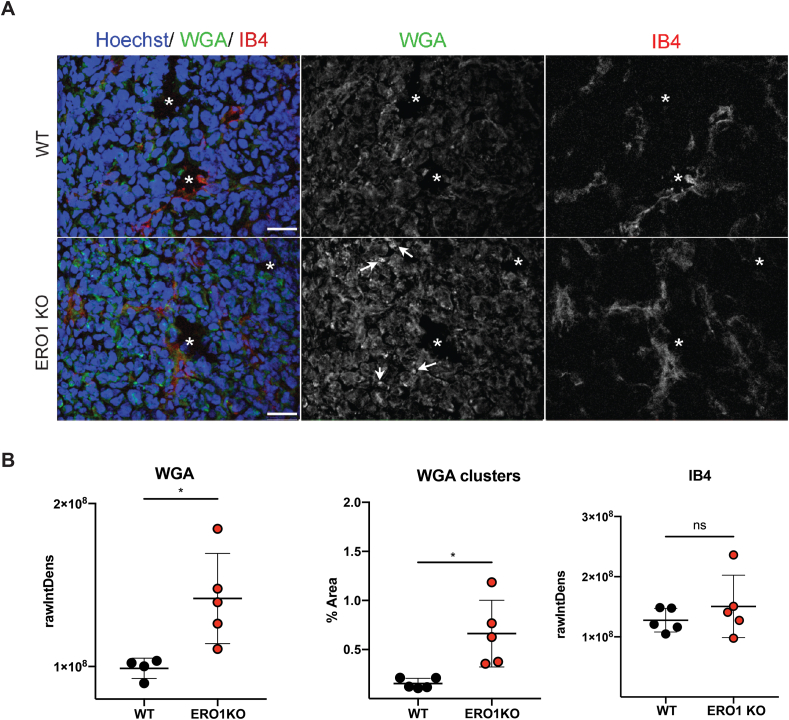
ERO1-KO breast tumors show a general pattern of N-hyper-glycosylated proteins A) Representative micrographs WGA, IB4 and Hoechst fluorescent staining in primary breast tumors (scale bars 30 μm). Asterisks indicate blood vessels and arrows indicate a few of the WGA clusters. B) Relative quantification of WGA fluorescence intensity, WGA clusters and IB4 fluorescence intensity (N = 5, *t*-test).

MAGT1 and STT3B depletion by siRNA caused hypoglycosylation of VEGF^121^ and, of VEGF^121^C68S in both WT and ERO1 KO cells, indicating that both VEGF^121^ and its mutant are substrates of STT3B/MAGT1 (Fig. 6E). However, N-glycosylation of VEGF^121^C68S, the mutant in the cysteine closest to the asparagine, was less sensitive to STT3B/MAGT1 interference. These findings indicate that ERO1 deletion potentiates the interaction between STT3B and the VEGF^121^ substrate *via* MAGT1, resulting in its N-hyper-glycosylation.

### 3.6 ERO1-devoid breast tumor xenografts show an altered glycosylation

To gain insight into the physiological relevance of protein N-hyperglycosylation mediated by ERO1 loss in tissues, we utilized a model that we recently created in our laboratory, consisting of triple negative breast tumor xenografts (MDAMB231) depleted or not of ERO1 [20]. Sections of these xenografts were stained with two lectins with different sugar specificity: wheat germ agglutinin (WGA) and Isolectin B4 (IB4). WGA binds sialic acid and N-acetylglucosaminyl-conjugated protein, the latter typical of N-glycoproteins, while IB4 binds terminal α-d-galactosyl- and N-acetyl-d-galactosamine, the latter a hallmark of O-glycosylated proteins [33]. While IB4 staining appeared quite similar in the two genetic-divergent tumors, WGA staining in ERO1 KO was on average one and half times higher than in WT cells (Fig. 7A and B and Fig. Sup. 6), and showed a patchy clustered pattern especially visible in the KO cells. Quantification of the volume occupied by these clusters revealed a five-fold increase in the KO versus WT tumors (Fig. 7B), indicating that ERO1 deficiency has a widespread role in regulating glycosylation.

## 4 Discussion

ERO1 is a protein disulfide oxidase of the ER that works as an intermediate catalyst, receiving electrons from PDI, which directly introduces disulfide bonds into newly synthesized proteins [11,34]. ERO1-mediated oxidative poise may also rely on the ability of this oxidase to generate stoichiometric amounts of H_2_O_2_, by the reduction of molecular O_2_, the final acceptor of this electron transport chain [13,35]. PRDX4, given its peroxidase activity, was able to metabolize ERO1-generated H_2_O_2_ and also to cope with ERO1 loss by substituting it in the relay of electrons with PDI, leading to PDI re-oxidation for a new cycle of disulfide bond formation in substrate proteins [14,16]. However, ERO1 loss of function is not fully compensated by PRDX4 and other oxidases: indeed, in previous work, we demonstrated a selective impairment of VEGFA-mediated angiogenesis in ERO1-devoid breast cancer under hypoxic conditions [20]. Here, with the goal of elucidating the effect of ERO1 loss on VEGFA secretion and angiogenic activity we have analyzed the post-translational modifications of a proangiogenic isoform of VEGFA, VEGF^121^.

In agreement with previous work [28], we find that ERO1 loss impaired the ER oxidative poise and show that this alteration slows down the formation of functional disulfide bonds in VEGF^121^. Nevertheless, mass spectrometric analysis of secreted VEGF^121^ together with functional SPR analysis of its interactions with VEGF-R1 and -R2 receptors ruled out massive aberrations in intramolecular disulphide bonds of VEGF^121^ from ERO1 KO cells suggesting that ERO1’s role in the oxidative protein folding is well compensated under normoxic conditions. Differently, under hypoxia, which selectively impairs the formation of post-translational disulfide bonds [32], VEGF^121^ secretion from ERO1 KO cells was severely reduced, suggesting a lack of compensation of ERO1 activity as oxidase under hypoxia.

The most remarkable finding of our study concerns the N-glycosylation of VEGF^121^ at N75 within the NIT sequon. Because its position within the sequence and because it is bracketed by a disulphide bridge, N75 is predicted to be a post-translational STT3B substrate [8]. We found that ERO1 deficit results in a more efficient utilization of this residue, and that both VEGF^121^C60S and C68S, which lack respectively an intermolecular bond and intramolecular disulfide bond, become hyper-glycosylated, indicating that the lack of these disulfides improves the ability of OST to glycosylate the N75 sequon. Furthermore, unbiased mass-spectrometric analysis of VEGF^121^ interactors and the consequent pathway analysis highlighted the pathway of Asparagine N-glycosylation, including the thioredoxin-like MAGT1 subunit of STT3B-containing OST, in ERO1 KO, but not in WT cells.

**Fig. 8:**
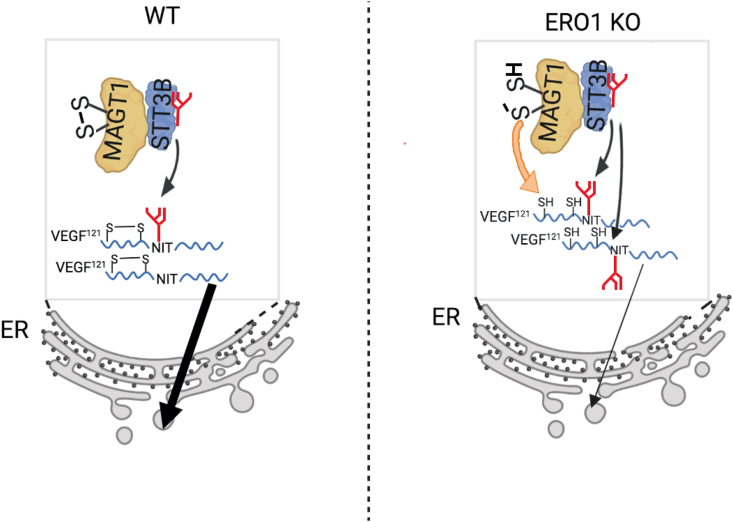
Working model ERO1 loss imposes less oxidizing ER redox poise. This results in a delay in disulfide bond formation in VEGF^121^, which favors its N-hyperglycosylation and increases MAGT1 trapping potential, further increasing its glycosylation on N75. This, in turn slows secretion (thin, versus thick arrow depicted in WT cells) of the growth factor in ERO1-depleted cells.

The interference of disulfide bonds with N-glycosylation of VEGF^121^ are in line with the structural studies showing the inaccessibility of the catalytic site of the bacterial OST homolog to the folded protein domains and with previous work demonstrating that cytosolic reducing poise improves STT3B activity [10,36]. The unanticipated finding of the current study is that the less oxidizing ER redox poise caused by ERO1 loss, while allowing the secretion of correctly assembled disulphide bonds in VEGF^121^, nevertheless alters their N-glycosylation. We suggest that the limited utilization of VEGF^121^'s acceptor site occurring in WT cells is based on the competitive kinetics between glycosylation and disulfide bridge formation: if the latter are formed rapidly, the substrate is denied access to OST (Fig. 8, left). In ERO1-depleted cells disulfide bond formation is slowed, thus giving OST more time for transfer of the high mannose oligosaccharide to the not yet oxidized protein. Furthermore, lack of ERO1 increases the interaction between VEGF^121^ and MAGT1, likely reinforcing the trapping potential of the latter in virtue of the more reduced (less oxidizing) ER poise, thereby reinforcing utilization of VEGF^121^'s acceptor site (Fig. 8, right). Consistent with this, the trapping mutant of MAGT1 can form stable mixed disulfides with STT3B substrate proteins [8]. Importantly, N-hyperglycosylation of VEGF^121^ in ERO1 KO has functional consequences, as we observe here that the secretion kinetics of hyperglycosylated VEGF^121^ together with its unglycosylated form are reduced. Similarly, N-hyperglycosylated VEGF^121^C68S is less secreted from WT cells suggesting a potential intracellular binding between the unglycosylated and N-hyperglycosylated VEGF^121^ and a consequent degradation. Although the underlying mechanism of this phenomenon remains to be elucidated, it likely accounts for the reduced migration and metastatic properties of ERO1 KO cells reported by us in a previous publication [20].

In addition to shedding light on the post-translational modifications and secretion of VEGF, a key protein in physiology and pathology, our study may have wider implications, as the ERO1 depletion might affect other N-glycosylated proteins, too. Indeed, staining of ERO1 KO breast tumors with the lectin WGA resulted in an increased, clustered signal which could be consistent with increased N-glycosylation in these tumors. In this respect, our results may be particularly relevant to cancer pathomechanisms, given that alterations in protein glycosylation impair cell growth, promote tumor-associated immune escape and metastasis, and inhibition of protein glycosylation has been recently proposed as a pro-angiogenic strategy [[37], [38], [39]]. Future studies will both shed light on the role of ERO1 in the complex interplay between disulfide bridge formation, N-glycosylation, and secretion, as well as open new avenues of research on the role of this oxidase in pathological processes.

## Authors’ contributions

EV, A Chernorudskiy and A Cherubini ran the experiments, AB and A Catteneo did mass-spectrometry and analyzed the related data, GE and MG obtained and analyzed SPR data, SF analyzed data of the ratiometric roGFP2, ML critically analyzed the experiments and provided reagents, NB critically analyzed the experiments and wrote the manuscript, EZ acquired funding, designed and oversaw the experiments, and wrote the manuscript.

## Declaration of competing interest

The authors declare no conflicting interests.

## Data Availability

Data will be made available on request.
