# Supplementary material for: ERO1 alpha deficiency impairs angiogenesis by increasing N-glycosylation of a proangiogenic VEGFA
Source: Redox Biol. 2022 Aug 27;56:102455. doi: 10.1016/j.redox.2022.102455 (PMC9463388; doi:10.1016/j.redox.2022.102455)

ERO1 alpha deficiency impairs angiogenesis by increasing N-glycosylation of a proangiogenic VEGFA

Ersilia Varone[1], Alexander Chernorudskiy[1*], Alessandro Cherubini[1], Angela Cattaneo[2], Angela Bachi[3], Stefano Fumagalli[1], Gizem Erol[1], Marco Gobbi[1], Michael J. Lenardo[4], Nica Borgese[5] & Ester Zito[1,6]

[1] Istituto di Ricerche Farmacologiche Mario Negri IRCCS, Milan, Italy.

[2] Proteomics/MS Facility, Cogentech SRL Benefit Corporation, Milan, Italy

[3] IFOM-FIRC Institute of Molecular Oncology, Milan, Italy

[4] Molecular Development of the Immune System Section, Laboratory of Immune System Biology, and Clinical Genomics Program, Division of Intramural Research, National Institute of Allergy and Infectious Diseases, National Institutes of Health, Bethesda, MD, USA.

[5] CNR Neuroscience Institute, c/o University of Milano Bicocca Vedano al Lambro (MB), Italy

[6] Department of Biomolecular Sciences, University of Urbino Carlo Bo, Italy.

[*]EV and AC contributed equally

Corresponding author:
Ester Zito,
ORCID ID: 0000-0001-7786-7698
Istituto di Ricerche Farmacologiche Mario Negri IRCCS
Via Mario Negri 2,
20156 Milano, Italy
Tel: +39 0239014480
E-mail: ester.zito@marionegri.it

Supplementary material and methods

Confocal ratiometric microscopy

The pcDNA3 roGFP2 plasmid encoding the ratiometric sensor SS_FLAG_roGFP2 (where SS is an artificial signal sequence) under the control of a CMV promoter was a kind gift from David Ron. RoGFP2- transfected cells were analysed by confocal ratiometric microscopy on a Nikon A1 confocal scan unit with a 40x objective at 1.49 zoom, managed by NIS elements software. Images at 512x512 pixels were obtained using laser excitation of 405 or 488 nm and emission light collected with a 525/50 nm filter, with a sequential scanning mode to avoid bleed-through effects. Random fields per condition were acquired longitudinally. Image analysis was done using the Ratio Plus ImageJ plugin. Briefly, cells at all time points were automatically segmented using an originally developed ImageJ-algorithm to define the regions of interest for quantification. For each channel, namely $Ex_{405}Em_{525}$ and $Ex_{488}Em_{525}$, we normalized background noise and applied a correction factor so to have an intensity ratio $Ex_{405}Em_{525}/Ex_{488}Em_{525}$ approximated to 1 at baseline. The same correction factor was applied to calculate channel intensity ratio after background normalization at subsequent time points as in (Chernorudskiy et al., 2020).

$VEGF^{121}$ expression constructs and mutagenesis

$VEGF^{121}$ sequence encoding the mature protein without the signal peptide (starting from Ala27) was PCR amplified from a human cDNA library (HeLa cells) with specific primers appending the restriction sites for HindIII and KpnI. The product was subcloned into an in-house produced expression vector derived from pFLAG-CMV-1 (Sigma-Aldrich), containing the sequence for

optimized signal peptide (SP; MSALLILALVGAAVA) followed by FLAG tag, producing the SP-FLAG-VEGF^{121} construct.

We used QuikChange II XL kit (Agilent) for single amino acid mutagenesis and the primers listed below. All the constructs were verified by Sanger sequencing.

**Primer**	**Sequence 5'-3'**
VEGF121, forward	GCACATAAGCTTGCACCCATGGCAGAAGGAGG
VEGF121, reverse	ATCCGGGGTACCTCACCGCCTCGGCTTGTCAC
VEGF C60S, forward	CGTCATTGCAGCTGCCCCCGCATCG
VEGF C60S, reverse	CGATGCGGGGGCAGCTGCAATGACG
VEGF C68S, forward	CAGTGGGCACACTCTCCAGGCCCTC
VEGF C68S, reverse	GAGGGCCTGGAGAGTGTGCCCACTG
VEGF N75Q, forward	CATAATCTGCATGGTGATCTGGGACTCCTCAGTGGGCAC
VEGF N75Q, reverse	GTGCCCACTGAGGAGTCCCAGATCACCATGCAGATTATG

PNGase F and Endo H treatment

All enzymes and reagents were purchased from New England Biolabs. Glycoprotein digestions with PNGase F and Endo H were done according to the manufacturer's manual. Briefly, protein samples were denatured by addition of 10x glycoprotein denaturation buffer (B1704) and heating at 99°C for 10 min. The denatured samples were supplemented with either 10x GlycoBuffer 3 (B1720) and Endo H (P0702; ~ 1 unit per ug of total protein), or 10x GlycoBuffer 2 (B3704), 1% NP-40 (B2704) and PNGase F (P0704; ~ 1 unit per ug of total protein). The reactions were incubated at 37°C for 1 h, then mixed with 4x Laemmli buffer for loading on SDS-PAGE gels.

Surface Plasmon Resonance (SPR)

1.2 x10^{6} WT and ERO1 KO cells were transfected with FLAG-VEGF^{121} or mutants, cultured in 8 mL of 1% FBS and high-glucose DMEM; the conditioned media were collected after 36 h quenched with 20mM NEM and purified with PD10 desalting columns (GE Healthcare).

Aflibercept (Regn3; 150 mg/mL), provided by Regeneron Pharmaceuticals, Inc. (Tarrytown, NY 10591-6707, USA) and B20.4.1.1 (B20; 250 ng/uL) by Genentech, Inc. (South San Francisco, USA), was captured on the SPR sensor chip as previously described (Papadopoulos et al., 2012) and then conditioned media containing FLAG-VEGF^{121} was fluxed and the affinity calculated under reference conditions.

SPR analyses were carried out using a Proteon XPR36 system (Bio-Rad Laboratories), which has six parallel flow channels that can immobilize up to six ligands on the same sensor chip. Protein A (Pierce, Rockford, IL) was immobilized first in two parallel strips of alginate hydrogel (AL) coated chip (XanTec Bioanalytics GmbH, DE) using amine coupling chemistry (Beeg et al., 2019). One of the two strips was used to capture Aflibercept. For this Aflibercept, 30 μg/mL in HBS-T (10 mM Hepes, 150 mM NaCl, 3.4 mM EDTA, 0.005% Tween 20, pH 7.4) was flowed over immobilized protein A for 10 min at a flow rate of 30 μL/min, resulting in ~5000 Resonance Units (RU, where 1 RU = 1 pg protein/mm^2) captured in a pseudo-irreversible manner. The parallel strip with immobilized Protein A (~6000 RU) was considered the reference surface. In another parallel strip we immobilized the anti-VEGF antibody B20 using amine coupling chemistry.

After ligand immobilization/capture, the ProteOn XPR36 fluidic system can rotate by 90° (Beeg et al., 2019) so that up to six different analytes, i.e. the conditioned media containing VEGF^{121}, can be injected simultaneously over all ligands (B20, Aflibercept and Protein A as reference). All SPR assays were run at a rate of 30 μL/min at 25°C. The sensorgrams (time course of the SPR signal in RU) were corrected for non-specific binding as recorded in the reference channel, and normalized to a baseline value of zero.

The relative concentrations of VEGF^{121} in the different conditioned media injected were estimated from the fitting of the sensorgrams obtained on B20, assuming the same binding constants (association and dissociation rate constants). These concentrations were then used for fitting the sensorgrams obtained on Aflibercept, in order to evaluate differences of the affinities of secreted VEGF^{121}.

MS Data analysis

Proteins were identified processing Raw files with Proteome Discoverer (version 1.4, Fischer Scientific). MS/MS spectra were searched with Mascot engine (version 2.6.0, Matrix Science) against the database uniprot_cp_human_2020 adding the sequences of $VEGF^{121}$, $VEGF^{121}$C60S, $VEGF^{121}$C68S, setting the parameters: enzyme Trypsin or Trypsin + GluC; max missed cleavage 2; variable modifications Carbamidomethylation (C), Oxidation (M), protein N-terminal Acetylation, N-ethylmaleimide (C), N-ethylmaleimide + water (C) and Deamidation (N); peptide mass tolerance 10 ppm; MS/MS tolerance 20 mmu. Scaffold (version Scaffold_4.3.3, Proteome Software Inc., Portland, OR) was used to validate MS/MS based peptide and protein identifications. Peptide identifications were accepted if they could be established at greater than 95.0% probability by the Scaffold Local FDR algorithm. Protein identifications were accepted if they could be established at greater than 99.0% probability and contained at least 2 identified peptides. Protein probabilities were assigned by the Protein Prophet algorithm. Raw data were also processed with MaxQuant version 1.6.0.16. Peptides were identified from the MS/MS spectra searched against the Data Bases uniprot_cp_human_2020 adding the sequences of $VEGF^{121}$, $VEGF^{121}$C60S, $VEGF^{121}$C68S, using the Andromeda search engine, in which GluC and Lys-C specificity were used with up to two missed cleavages allowed. Cysteine carbamidomethylation, N-ethylmaleimide and N-ethylmaleimide+water, Metionine oxidation, protein N-terminal Acetylation and Asparagine deamidation were used as variable modifications. The peptide and protein false discovery rates (FDRs) were set to 0.01 and mass deviation for MS and MS/MS peaks was set at 10 and 20 ppm respectively. The minimal length required for a peptide identification was six amino acids.

Raw files of the proteomic data together with the parameters used for the analysis have been deposited into PRIDE.

Protein in-gel digestion and mass spectrometry analysis

Proteins were FLAGM1-immunoprecipitated from lysates of FLAG-VEGF[121] transfected cells. The proteins were then resolved on reducing and non-reducing 15% SDS-PAGE gels and Coomassie stained. For identification of VEGF[121] interactors, sections of gel from 270 kDa to 30 kDa were excised, reduced with 10 mM DTT, alkylated with 55 mM NEM and digested overnight with trypsin. Acidified peptide mixtures were desalted and concentrated on StageTipC18 (Rappsilber et al., 2003), and injected as technical replicates on a nLC-ESI-MS/MS quadrupole Orbitrap QExactive-HF mass spectrometer (Thermo Fisher Scientific).

The peptides were separated on a linear gradient from 95% Solvent A (0.1% formic acid, 2% acetonitrile) to 50% Solvent B (80% acetonitrile, 0.1% formic acid) over 23 min and from 50% to 100% Solvent B in 2 min at a constant flow rate of 0.25 µl min−1 on a UHPLC Easy-nLC 1200 (Thermo Scientific). The LC system was connected to a 25 cm fused-silica emitter of 75 µm inner diameter (New Objective), packed in house with ReproSil-Pur C18-AQ 1.9 µm beads (Dr. Maisch) using a high-pressure bomb loader (Proxeon). MS data were acquired in positive mode using a DDA top15 method for HCD fragmentation. Survey full scan MS spectra (300–1750 Th) were acquired in the Orbitrap with 60,000 resolution, AGC target 1e6, IT 120 ms. For HCD spectra the resolution was set to 15,000, AGC target 1e5, IT 120 ms; normalized collision energy 28%, isolation width 3.0 m/z and dynamic exclusion of 5 sec.

For the analysis of cysteine modifications, bands corresponding to dimers and oligomers of secreted VEGF[121] were excised and treated with 55 mM NEM to alkylate the reduced cysteines, then the disulfide bonds were reduced with 10 mM DTT and alkylated with 55 mM iodoacetamide (IAA).

For the analysis of N-glycosylation, after cysteine alkylation, samples were digested with 750U PNGaseF (BioLabs) and deamidated proteins were double digested by GluC and Lys-C or Trypsin. Acidified peptide mixes were treated and analyzed by nLC-ESI-MS/MS as described above.

References:

Beeg, M., A. Nobili, B. Orsini, F. Rogai, D. Gilardi, G. Fiorino, S. Danese, M. Salmona, S. Garattini, and M. Gobbi. 2019. A Surface Plasmon Resonance-based assay to measure serum concentrations of therapeutic antibodies and anti-drug antibodies. *Sci Rep*. 9:2064.

Chernorudskiy, A., E. Varone, S.F. Colombo, S. Fumagalli, A. Cagnotto, A. Cattaneo, M. Briens, M. Baltzinger, L. Kuhn, A. Bachi, A. Berardi, M. Salmona, G. Musco, N. Borgese, A. Lescure, and E. Zito. 2020. Selenoprotein N is an endoplasmic reticulum calcium sensor that links luminal calcium levels to a redox activity. *Proc Natl Acad Sci U S A*. 117:21288-21298.

Papadopoulos, N., J. Martin, Q. Ruan, A. Rafique, M.P. Rosconi, E. Shi, E.A. Pyles, G.D. Yancopoulos, N. Stahl, and S.J. Wiegand. 2012. Binding and neutralization of vascular endothelial growth factor (VEGF) and related ligands by VEGF Trap, ranibizumab and bevacizumab. *Angiogenesis*. 15:171-185.

Rappsilber, J., Y. Ishihama, and M. Mann. 2003. Stop and go extraction tips for matrix-assisted laser desorption/ionization, nanoelectrospray, and LC/MS sample pretreatment in proteomics. *Anal Chem*. 75:663-670.

Supplementary figure legends

Figure Sup. 1

Uncropped non-reducing immunoblot of FLAG-VEGF^{121} immunoprecipitates (IP) extracted from WT and ERO1 KO HeLa cells; the boxes enclose the portions shown in the cropped version of Fig. 1C. All IP samples of ERO1 KO cells were loaded twice in volume than WT to render the otherwise faint bands of VEGF^{121} more clear and quantifiable.

Figure Sup. 2

A) Coomassie stained reducing and non-reducing SDS-PAGE of FLAGM1-immunopurified VEGF^{121} secreted from WT and ERO1 KO cells. The uncropped gel, containing the lanes shown in Fig. 2A is shown on the right. The gel on the right includes also two control samples (Co), i.e., FLAGM1 immunoprecipitates from lysate of cells transfected with an empty vector. On the left, Coomassie stained in reducing and non-reducing SDS-PAGE of FLAGM1-immunopurified VEGF^{121} secreted from WT and ERO1 KO cells, on the right part is indicated the size of the monomer (in reducing conditions), on the left the size of dimer and oligomer (in non-reducing conditions). B) Representative MS/MS spectrum of the secreted VEGF^{121} peptide containing the NIT N-glycosylation consensus (see Fig. 2D) after PNGaseF treatment. The peptide is shown in the deamidated and amidated state.

Figure Sup. 3

A) Ponceau Staining of Fig. 3A. B) Uncropped reducing immunoblot of FLAG-VEGF^{121} and N75 mutant corresponding to the cropped version (boxed) of Fig. 3B. C) A) Ponceau Staining of Fig. 3C.

Figure Sup. 4

A) Uncropped Coomassie stained reducing SDS-PAGE of FLAGM1-immunopurified VEGF^{121}, VEGF^{121}C60S and C68S in WT and ERO1 KO cells. The boxes enclose the portions shown in the cropped version in Fig. 5C. B) Representative MS/MS spectrum of the intracellular VEGF^{121} peptide containing the NIT N-glycosylation consensus after PNGaseF treatment. The peptide is shown in the deamidated and amidated state.

Figure Sup. 5

Uncropped reducing immunoblots of FLAG and MAGT1 of FLAGM1-immunopurified VEGF^{121} and C68S in WT and ERO1 KO cells corresponding to the cropped version (boxed) of Fig. 6 C. MAGT1 panel was probed with two different antibodies (lower two panels) with similar results.

Figure Sup. 6

Representative micrographs of negative control staining of WT and ERO1 KO MDAMB231 breast tumors where the lectins WGA and IB4 were omitted (scale bar 30 μm). Hoechst stains nuclei (blue). Asterisk indicates blood vessels.

Figure Sup.1

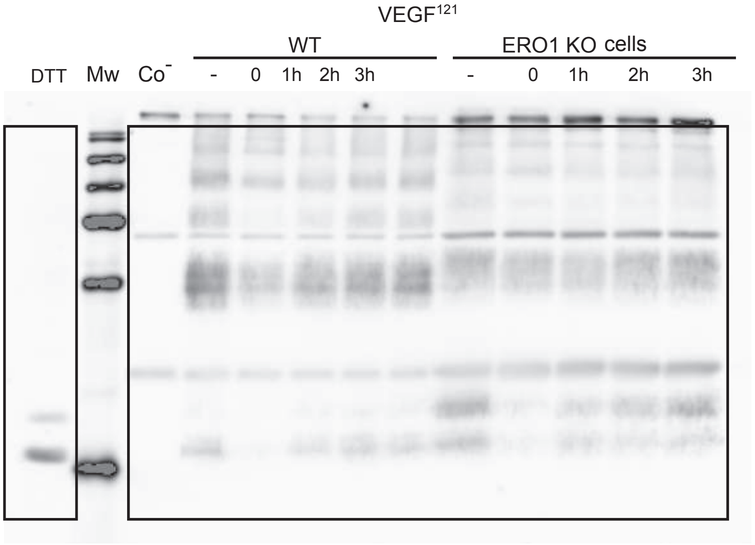

Figure Sup.2

A

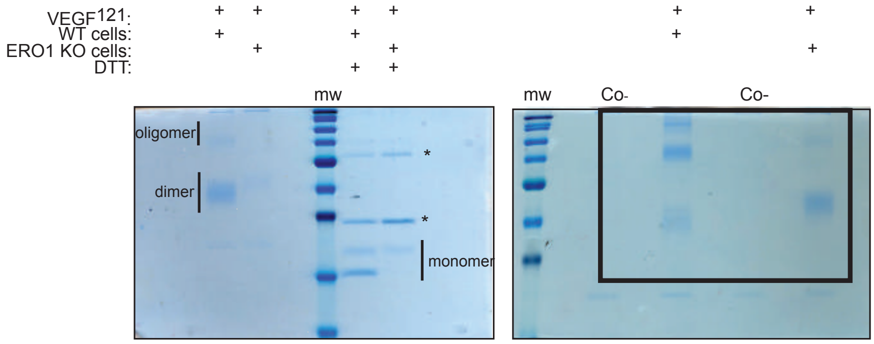

B

m/z= 784.07, 3+ Gene name = VEGF121 peptide SNITM(Oxidation +16)QIM(Oxidation +16)RIKPHQGQHIGE

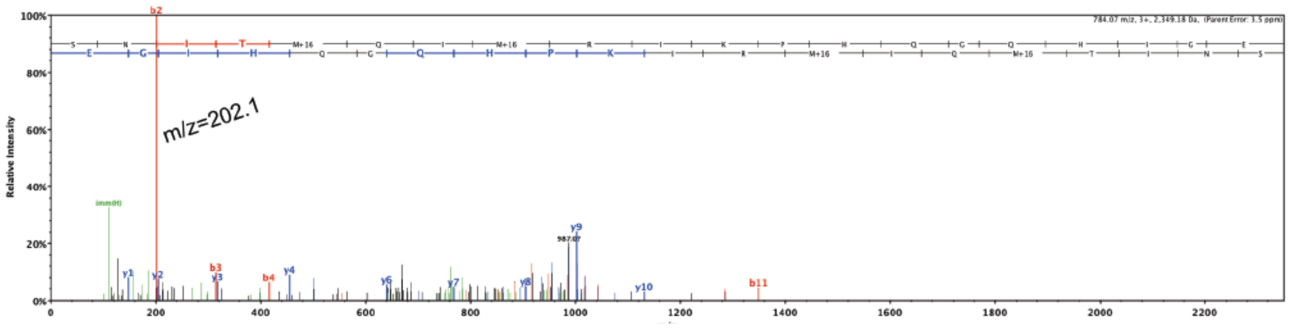

m/z= 779.06, 3+ Gene name = VEGF121 peptide SN(Deamidated +1)ITMQIM(Oxidation +16)RIKPHQGQHIGE

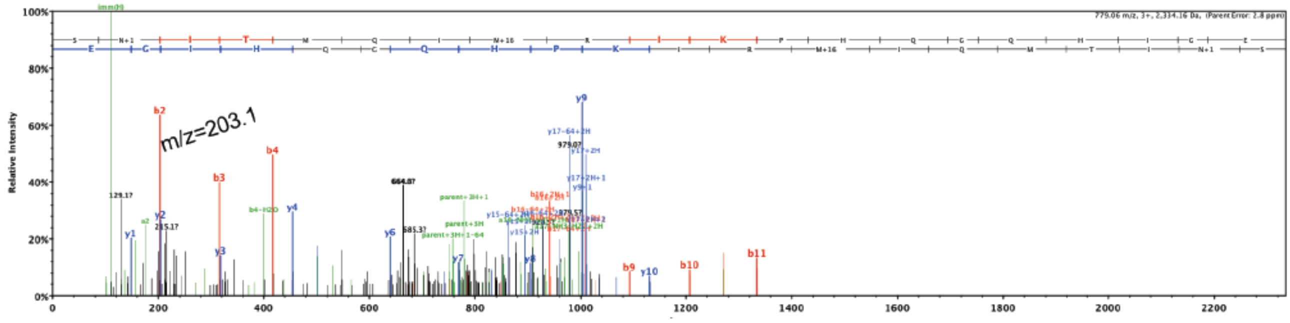

Figure Sup.3

A

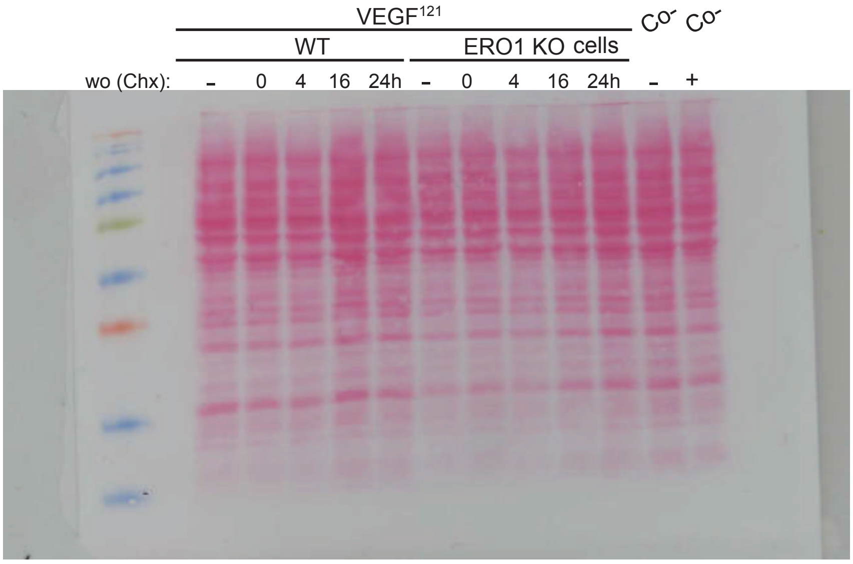

B

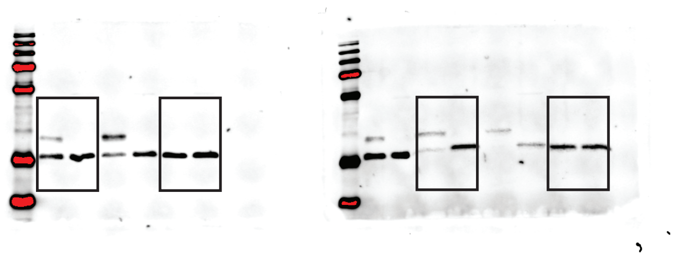

C

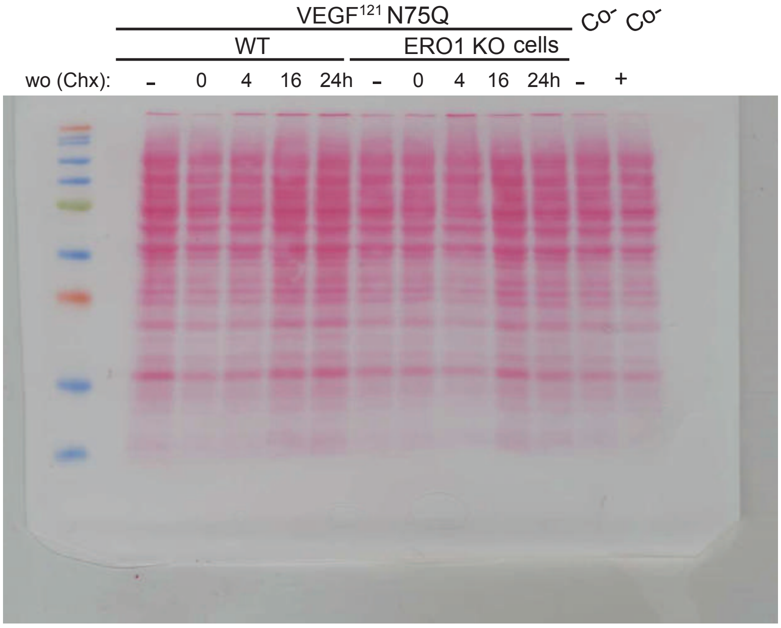

Figure Sup.4

A

VEGF^{121} VEGF^{121}C68S VEGF^{121}C60S Co Mw

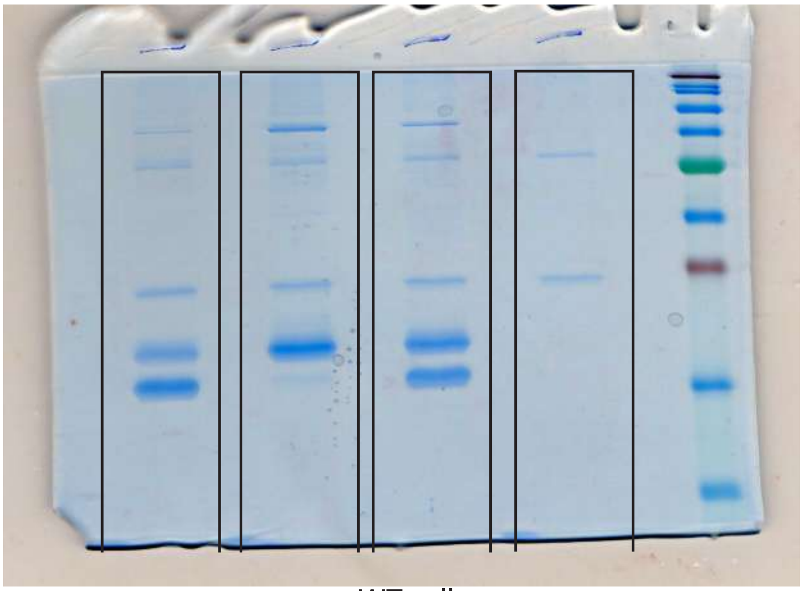

WT cells

VEGF^{121} VEGF^{121}C68S VEGF^{121}C60S Co Mw

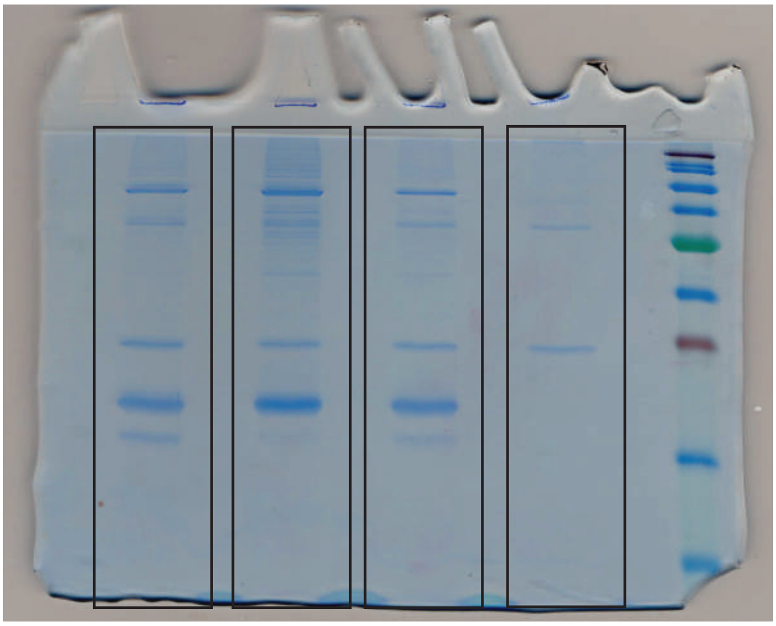

ERO1 KO cells

B

m/z= 547.28, 2+ VEGF^{121} peptide SNITMQIMR

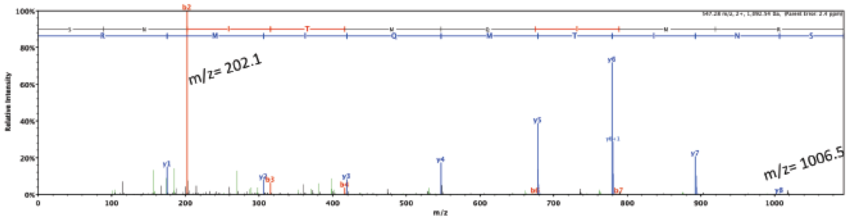

m/z= 547.77, 2+ VEGF^{121} peptide SN(Deamidated +1)ITMQIMR

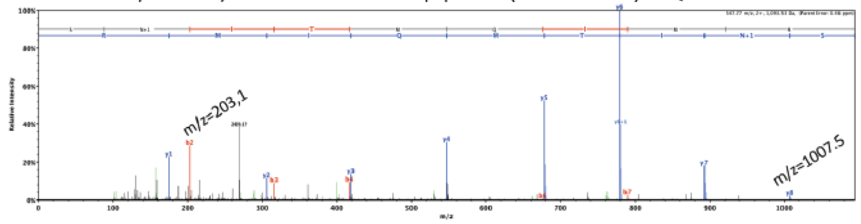

Figure Sup.5

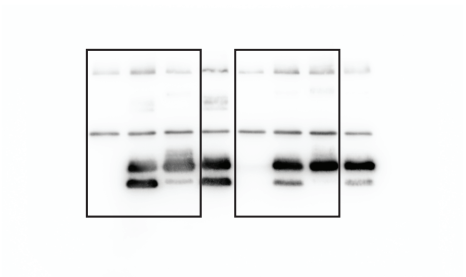

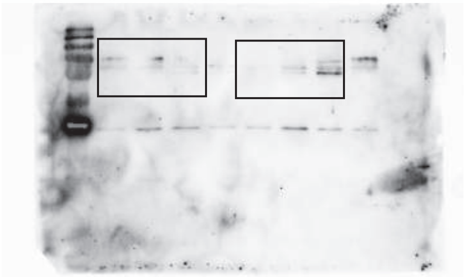

MAGT1

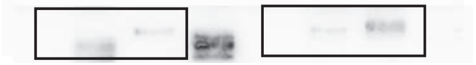

MAGT1

Figure Sup. 6

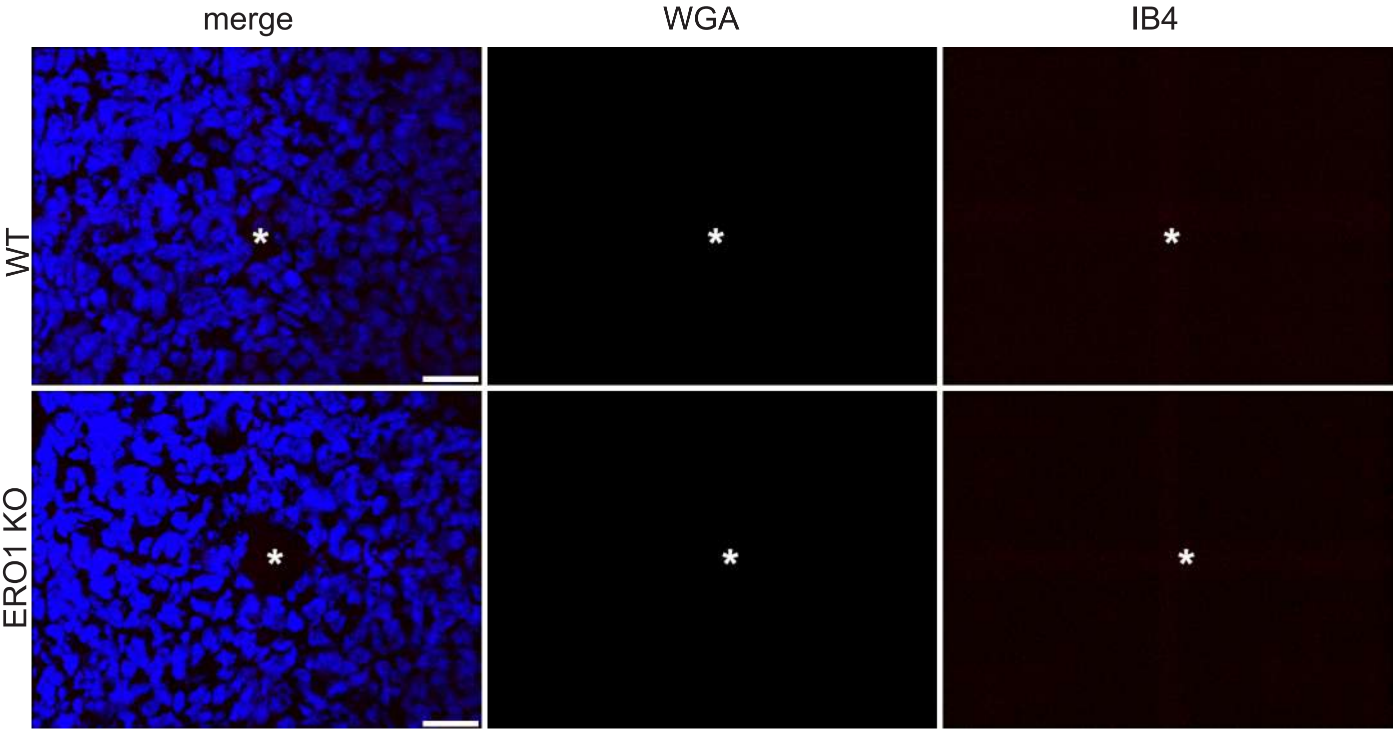

Supplement: Multimedia component 1 [file mmc1.pdf]
